# c-Myc Accelerates S-Phase and Requires WRN to Avoid Replication Stress

**DOI:** 10.1371/journal.pone.0005951

**Published:** 2009-06-18

**Authors:** Kristin Robinson, Nichaya Asawachaicharn, Denise A. Galloway, Carla Grandori

**Affiliations:** Program in Cancer Biology and Division of Human Biology, Fred Hutchinson Cancer Research Center, Seattle, Washington, United States of America; Roswell Park Cancer Institute, United States of America

## Abstract

c-Myc interacts with components of the pre-replication complex and directly regulates DNA replication [1]. However the consequences of this novel c-Myc function on cell cycle dynamics and replication-associated damage are unknown. Here, we show that c-Myc overexpression in primary human fibroblasts markedly accelerates S-phase while c-Myc deficient fibroblasts exhibit a prolonged S-phase. We also show that the Werner DNA helicase protein (WRN) plays a critical role in supporting c-Myc-driven S-phase, as depletion of WRN in c-Myc overexpressing cells increases DNA damage specifically at sites of DNA synthesis. This excess DNA damage activates a “replication stress” pathway involving ATR, CHK1, CHK2, and p53, leading to rapid senescence of WRN deficient c-Myc overexpressing cells. Indeed, depletion of p53 rescues this senescence response. We propose that WRN functions to repair abnormal replication structures caused by the acceleration of DNA replication by c-Myc. This work provides an additional mechanistic explanation for c-Myc-induced DNA damage and senescence, and reveals a vulnerability of c-Myc overexpressing cells that could potentially be exploited in cancer therapy.

## Introduction

The profound effects of c-Myc on cell growth, proliferation, apoptosis, and tumorigenesis have been mainly attributed to its ability to coordinate gene transcription. c-Myc is a basic helix-loop helix transcription factor that directly modulates transcription of a large number of genes controlled by all three RNA polymerases [2], [3], [4]. c-Myc can also indirectly regulate transcription through up-regulation of histone acetylases, with the potential to globally influence chromatin structure [5]. In addition, previous studies have demonstrated that c-Myc overexpression triggers the generation of double strand breaks and thus promotes chromosomal instability [6], [7]. Several mechanisms have been proposed to explain this phenomena, including overriding checkpoints [8]. c-Myc interference with DNA repair mechanisms [9] and increased production of reactive oxygen species, (ROS) [10]. However, a recent study has identified a novel function for c-Myc in the direct stimulation of DNA replication, independent of its transcriptional targets [1], thus providing yet a different potential mechanism for c-Myc induced genomic instability. This new c-Myc function was demonstrated by its ability to bind to components of the pre-replication complex, both in mammalian as well as in *Xenopus* cells, and to influence the rate of DNA replication in *Xenopus* cell-free extracts [1], [11]. In addition, c-Myc binds to known mammalian replication origins [1]. We hypothesized that overexpression of c-Myc might accelerate S-phase, leading to increased sensitivity to replication stress. As c-Myc-expressing cells show impaired proliferation and rapid senescence in the absence of the Werner DNA helicase, we further hypothesize that WRN is required to minimize replication stress during c-Myc driven S-phase.

The *WRN* gene encodes for a RecQ-DNA helicase, which has been shown to resolve DNA structures that form during S-phase, such as those generated at stalled of replication forks [12], [13]. Loss of function mutations in *WRN* cause progeroid features in humans, a condition known as Werner Syndrome (WS) [14]. Fibroblasts isolated from WS patients exhibit genomic instability, increased sensitivity to specific DNA damaging agents, slow proliferation, lengthened S-phase, and accelerated replicative senescence [15], [16]. These phenotypes have been in part recapitulated by acute knock-down of WRN protein by RNAi [17], [12]. hTERT overexpression can immortalize WS fibroblasts, although it does not rescue their slow growth, or the S-phase defects ([18] and CG unpublished observations). These findings are consistent with a role for the WRN helicase in maintaining genome integrity during DNA replication, in addition to its described role in telomere maintenance and stability [19], [20].

Here we monitored the effect of c-Myc expression on the rate of S-phase in human foreskin fibroblasts (HFF) and in *c-myc* deficient rat cells. Our experiments demonstrate that c-Myc overexpression markedly accelerates S-phase and conversely, *c-myc* deficient cells exhibit a greatly prolonged S-phase. Furthermore, WRN plays a critical role in this phenotype, as depleting WRN in c-Myc overexpressing cells leads to an increase in DNA damage, and cellular senescence. These results provide a mechanism for c-Myc-induced senescence and identify the DNA replication-repair machinery as a potential therapeutic target for c-Myc overexpressing tumor cells.

## Results and Discussion

**Figure 1:**
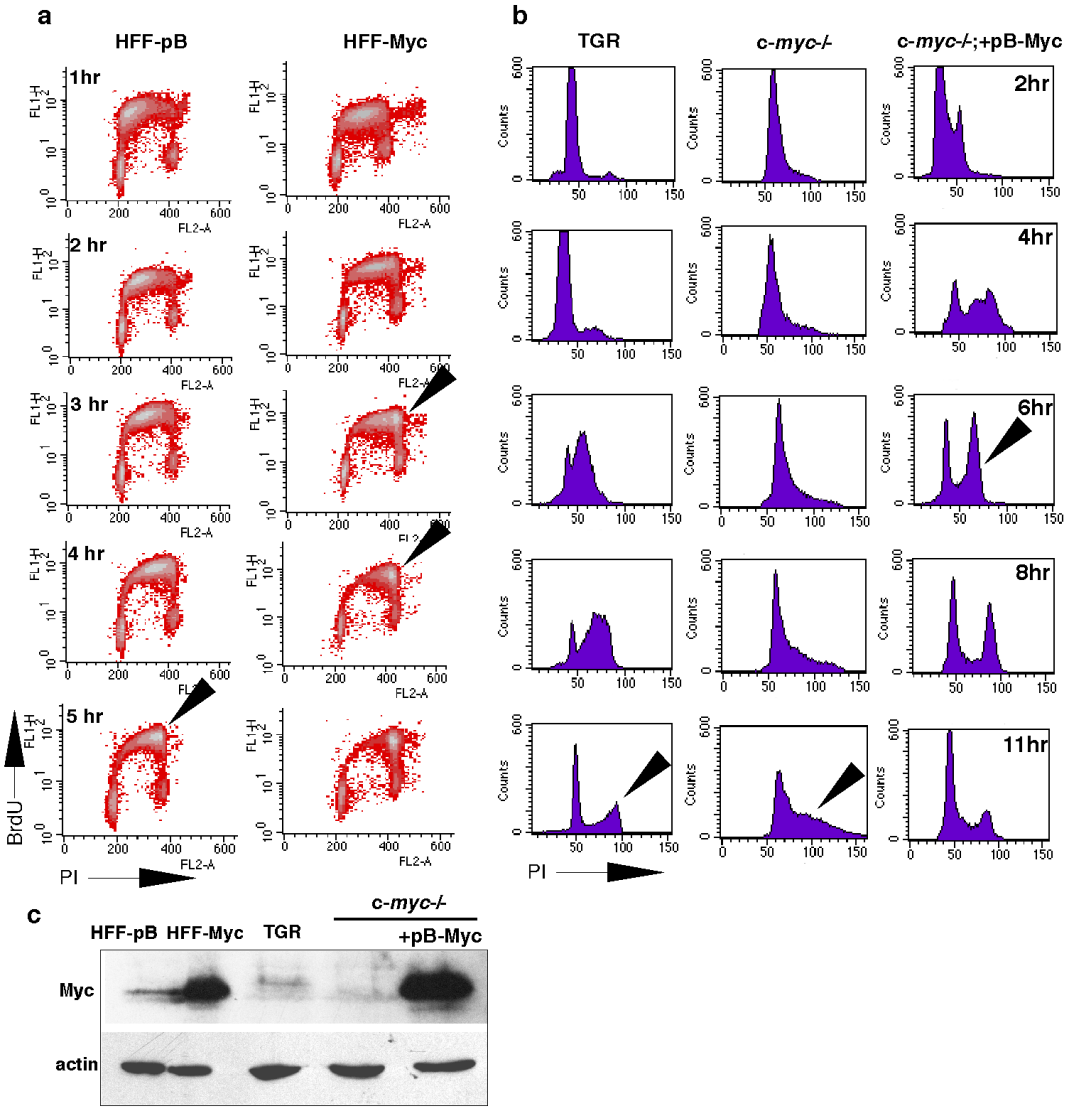
c-Myc levels determine the rate of S-phase. a) c-Myc accelerates S-phase in human foreskin fibroblasts (HFFs). BrdU pulse-chase experiment in HFFs transduced with pB-Myc retrovirus or empty vector, pBabe. Cells were synchronized by thymidine block and released in BrdU for one hour, then chased in BrdU-free media for the indicated time. Progression of early labeled BrdU cells through S and G2 was monitored by FACS analysis. The BrdU positive HFF-Myc cells accumulated in G2 between three to four hours after release, while the control cells HFF-pB accumulated in G2 beginning at five hours. Propidium iodide staining at the time of release, prior to BrdU addition to verify the G1/S arrest of HFF-Myc cells is shown in supplementary Figure S1. b) Knockout of *c-myc* lengthens S-phase. Propidium iodide profiles of the rat cell line TGR, its derivatives *c-myc*−/− [22] and the reconstituted *c-myc*−/−;+pB-Myc upon release from thymidine block is shown. While TGR cells completed S-phase between eight and eleven hours, *c-myc*−/− cells were still in S-phase at eleven hours. Reconstituted *c-myc*−/−;+pB-Myc cells showed a dramatic acceleration of S-phase (six hrs). c) Expression of c-Myc was analyzed by western blot. Actin is shown as loading control.

To examine the effect of c-Myc upon cell cycle dynamics we utilized primary human foreskin fibroblasts (HFF), as these cells tolerate c-Myc overexpression using a retroviral vector (pB-puro), and exhibit c-Myc-specific phenotypes, but do not develop the senescence response that is seen in other cell types [21]. HFF-Myc cells exhibit faster growth and increased size and prominent nucleoli, increased rRNA synthesis, and a global gene expression signature typical of c-Myc overexpressing cells [3] and CG unpublished). To examine the duration of S-phase, HFF-pB and HFF-Myc cells were synchronized at the G1/S boundary by double thymidine block. Upon release from G1/S, cells were labeled for one hour with BrdU and then chased for the indicated times and subjected to FACS analysis (Fig. 1). The progression of BrdU/propidium iodide-stained cells through S-phase into G2 as shown by the accumulation of cells with a G2 DNA content, indicated that HFF-pB cells completed S-phase within five to six hours, as expected for the duration of S-phase of mammalian cells (Fig. 1). In contrast, HFF-Myc cells showed a greatly reduced duration of S-phase, completing S-phase within three to four hours. This indicates that c-Myc overexpression accelerates the duration of S-phase by ∼40% in normal human fibroblasts. To determine if the loss of c-Myc slowed the duration of S-phase, we examined the *c-myc*−/− TGR rat cell line in comparison with the *c-myc* wild type TGR parental cell line [22]. After release from the G1/S block, the TGR parental line reached G2/M after eleven hours, at which time *c-myc*−/− cells were still in the middle of S-phase (Fig. 1b). An additional time course showed that the *c-myc*−/− cells completed S-phase at approximately fourteen hours from the time of release (data not shown), thus extending S-phase by three hours. In sharp contrast, the c-Myc-reconstituted knockout TGR cells, (*c-myc*−/−;+pB-Myc) cells had an accelerated S-phase completed at six hours (Fig. 1b). Thus, loss of c-Myc extended S-phase, and this delay was not just rescued by c-Myc overexpression, but in fact was accelerated nearly two fold relative to the wild type cells.

These data are consistent with the results obtained in HFFs, although they differ in two ways. First, a substantial fraction of *c-myc* reconstituted TGR line did not arrest at G1/S after thymidine block (see Fig. 1b, time 0 hour). Second, the parental TGR cells exhibited a prolonged arrest after thymidine release, leading to an apparently longer S-phase then the HFFs (eleven hours vs. six hours, Fig. 1a and b). Nevertheless, this experiment indicates that the levels of c-Myc can dramatically influence not only entry into S-phase as previously described [22], but also the duration of S-phase. These results, together with the previous observation that c-Myc overexpressing cells show an increased number of replication foci [1], suggest that c-Myc may increase the number of active replication origins, thus reducing replication timing.

**Figure 2:**
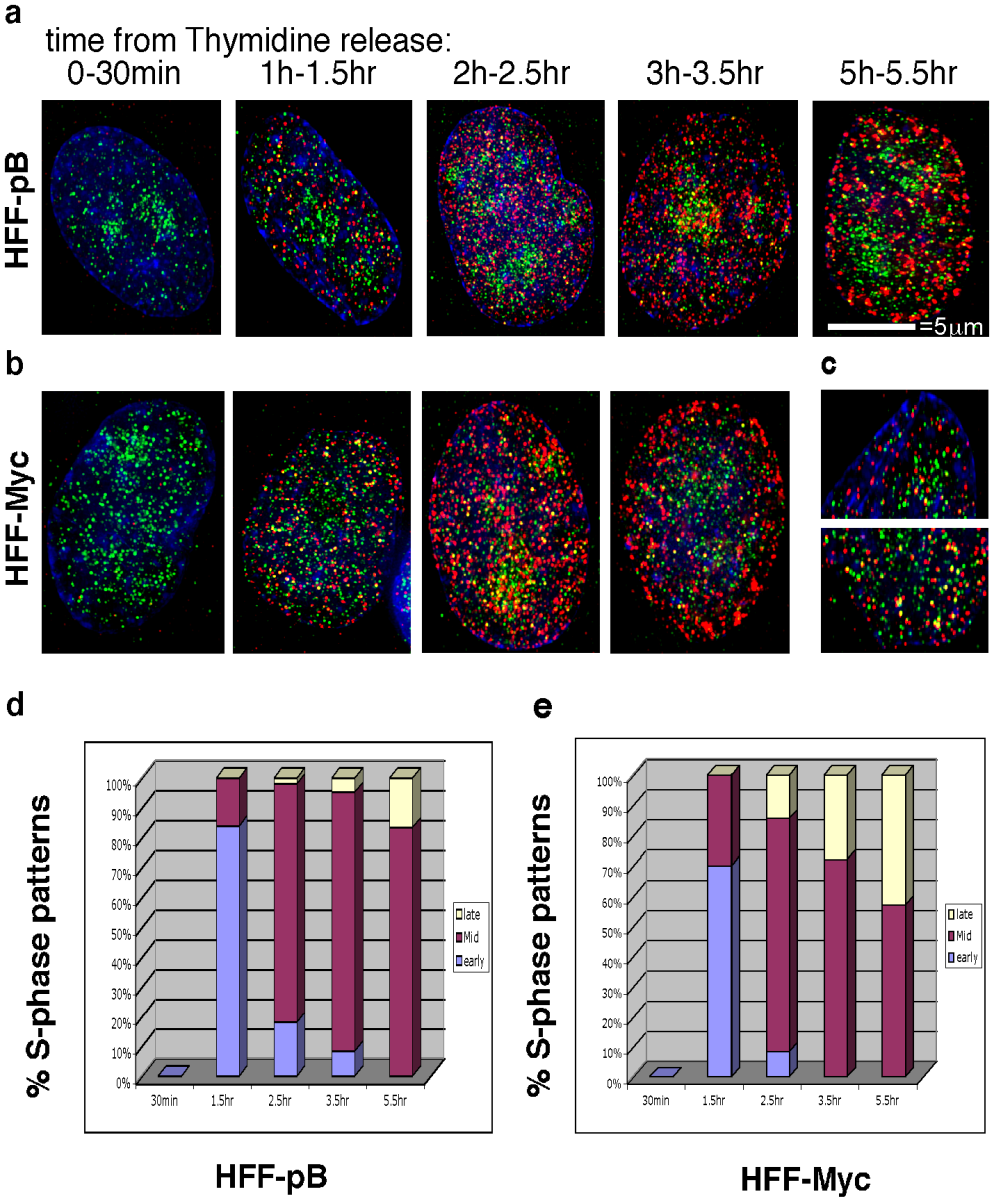
Increased presence of WRN at replication sites in c-Myc overexpressing HFFs. HFF-pB (a) and HFF-Myc (b) were synchronized by thymidine block, labeled at the indicated times for 30 minutes with BrdU, and fixed for staining. Cells were stained for WRN (green) and BrdU (red), and DNA was stained with Hoechst (blue). Images were collected with a 100X oil–immersion objective. An increased co-localization of WRN with BrdU foci (yellow) is detectable at the 1.5 and 2.5 hr time point in HFF-Myc cells. This indicates recruitment of WRN at sites of newly replicated DNA caused by c-Myc overexpression. A 3-D movie reconstructed from this experiment can be seen as supplemental data (Supplementary Movies S1 and S2). c) Higher magnification of HFF-pB (top) and HFF-Myc (bottom) cells at 1–1.5 hr time point to better visualize WRN and BrdU co-localized foci. d), e) Percentage of cells in early, intermediate, and late S-phase determined by the BrdU patterns. HFF-Myc (e) progressed faster than HFF-pB (d) and only “late” patterns were present at 3.5 hrs post-release, similar to the result obtained by the pulse-chase experiment (Fig. 1).

As WRN participates in the repair of abnormal DNA replication structures we next asked if WRN function was required to resolve replication-associated damage during c-Myc accelerated S-phase. We first examined the sub cellular distribution of WRN in relation to replication sites during S-phase (Fig. 2) using indirect immunofluorescence in G1/S synchronized HFF-pB and HFF-Myc cells. Cells were labeled for 30 minutes with BrdU at several time points after release from thymidine block. The WRN protein was mainly restricted to nucleoli in control fibroblasts (HFF-pB) as expected [23]. In contrast, WRN staining was most prominent at extra-nucleolar sites in HFF-Myc cells (compare 30 min. time point in Fig. 2a and b). The increase in overall WRN staining in HFF Myc cells is consistent with known effect of Myc on *WRN* gene transcription [24]. The abundance of extra-nucleolar WRN foci in HFF Myc cells suggested that WRN might be involved in repairing DNA damage during c-Myc driven S-phase, as DNA damage can cause re-localization of WRN from nucleoli to nucleoplasmic sites [25]. Co-localization of BrdU with WRN, both nucleolar and nucleoplasmic, was detected during middle to late S-phase in both HFF-pB and HFF-Myc cells (Fig. 2). However, WRN and BrdU co-localization were more frequently seen in HFF-Myc cells and appeared at earlier time points (compare one and two hour time points in Fig. 2a and b; enlarged in Fig. 2c and supplemental movies S1 and S2). The recruitment of WRN at sites of DNA replication indicated that WRN might be required to resolve abnormal replication structures during c-Myc-driven S-phase. The kinetics of BrdU staining also indicated that HFF-Myc cells moved faster through S-phase (Fig. 2b). This was evident by the quick substitution of early-S BrdU patterns [26] by intermediate and late S-phase patterns occurring two to three hours after thymidine release, with virtually no BrdU staining three and a half hours after release (Fig. 2b and quantified in 2e) indicating that HFF-Myc had completed S-phase within ∼three and a half hours. In contrast, HFF-pB control cells showed the expected BrdU labeling patterns [26] with late replicating cells appearing within four to six hours after thymidine release (Fig. 2a and 2d). These results confirmed that c-Myc overexpression accelerates transit through S-phase as also concluded from the BrdU pulse-chase experiments shown in Fig. 1.

**Figure 3:**
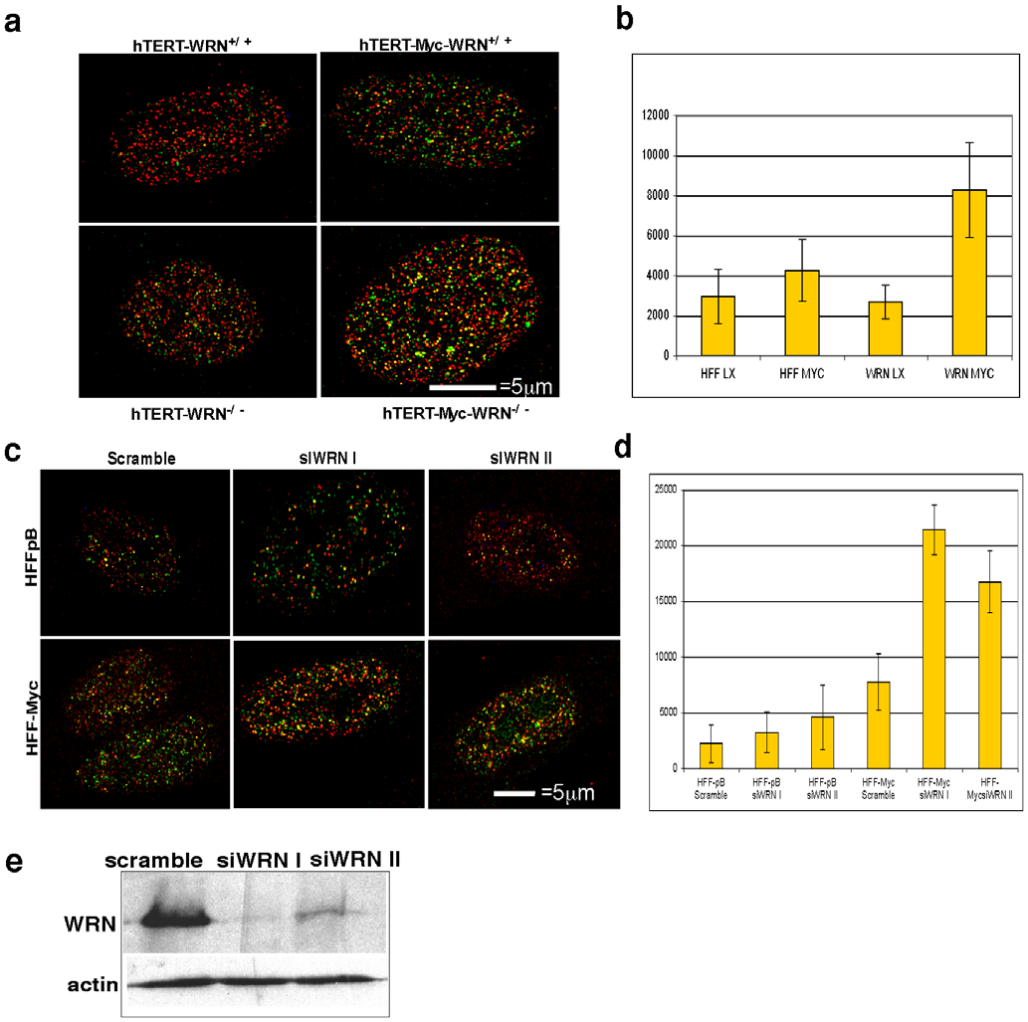
DNA damage accumulates at replication sites in WRN deficient cells in response to c-Myc overexpression. a) *hTERT*-immortalized WRN^−/−^ fibroblasts and normal *hTERT*-control fibroblasts were transduced with LXSN-Myc expressing retrovirus and labeled with BrdU for 30 minutes during S-phase. Cells were stained with anti-BrdU (red) and anti-γ-H2AX (green). Images were collected with a 100X oil–immersion objective. Note the increased appearance of γ-H2AX and BrdU spots (yellow) in WRN^−/−^ cells expressing c-Myc versus vector, LXSN, transduced WRN^−/−^ cells or WRN^+/+^ cells also expressing c-Myc. b) BrdU and γ -H2AX co-localized foci from (a) were counted in 10 cells/condition (Imaris software). c) HFF-Myc and HFF-pB cells were transduced with retroviruses encoding two different short hairpins to WRN, siWRN I and II, or scrambled control and labeled with BrdU at the first passage after infection. Knockdown using either siWRN virus caused increased co-localization of γ -H2AX (green) and BrdU (red). d) Quantitation of γ -H2AX and BrdU of experiment shown in (c). e) Western analysis of a parallel transduction of HFFs indicating the WRN knockdown achieved with siWRN I and II. Actin is shown as loading control.

Both the acceleration of S-phase in HFF-Myc cells and the presence of WRN at DNA replication sites prompted the question of whether loss of WRN function precipitates DNA damage during S-phase. We thus compared γ-H2AX foci; a marker of DNA damage, in WRN deficient cells that have been immortalized by h-TERT overexpression, (hTERT -WRN^−/−^) versus normal h-TERT immortalized human fibroblasts, (hTERT-WRN^+/+^) [24]. γ-H2AX foci coinciding with sites of newly replicated DNA were quantified by labeling cells with a short pulse of BrdU prior to fixation. The co-localization of BrdU and γ-H2AX was also compared in hTERT- WRN^+/+^ -fibroblasts versus hTERT-WRN^−/−^ fibroblasts after c-Myc overexpression with the c-Myc retroviral vector, LXSN-Myc. c-Myc expression in hTERT-WRN^−/−^ cells led to a 3.5-fold increase in γ-H2AX foci co-localizing with BrdU, relative to hTERT-WRN^−/−^ cells with empty vector (Fig. 3a and 3b), indicating c-Myc induced DNA damage occurs at sites of DNA replication. In contrast, only a 1.5-fold increase in BrdU/γ-H2AX foci was caused by LXSN-Myc in hTERT-WRN^+/+^ control fibroblasts (Fig. 3a and b), indicating that WRN loss allowed c-Myc-induced DNA damage to accumulate, presumably due to lack of repair. The number of γ-H2AX/BrdU foci is likely an underestimation of S-phase DNA damage, as cells were labeled with BrdU for only 30 minutes to capture DNA-damage associated with newly replicated DNA. Thus, BrdU negative γ-H2AX foci could also represent damage incurred during S-phase. BrdU labeling was only detectable in cells undergoing S-phase and not in thymidine arrested or quiescent cells, thus excluding the possibility of labeling replication-associated repair (see Fig. 2a, 30 min after thymidine release and data not shown). These results indicated that c-Myc-induced DNA damage occurred during S-phase and was aggravated in WRN deficient cells. To determine if acute depletion of WRN protein in normal HFFs also caused replication-associated damage, two WRN specific siRNAs were expressed from retroviral vectors (siWRN I and II; Fig. 3e). WRN knockdown synergized with c-Myc overexpression to aggravate DNA-replication associated damage as shown by increased γ-H2AX/ BrdU foci (Fig. 3c and quantitated in Fig. 3d). These results confirm that both acute and chronic WRN deficiency exacerbates the accumulation of DNA damage at replication sites under conditions of c-Myc overexpression.

**Figure 4:**
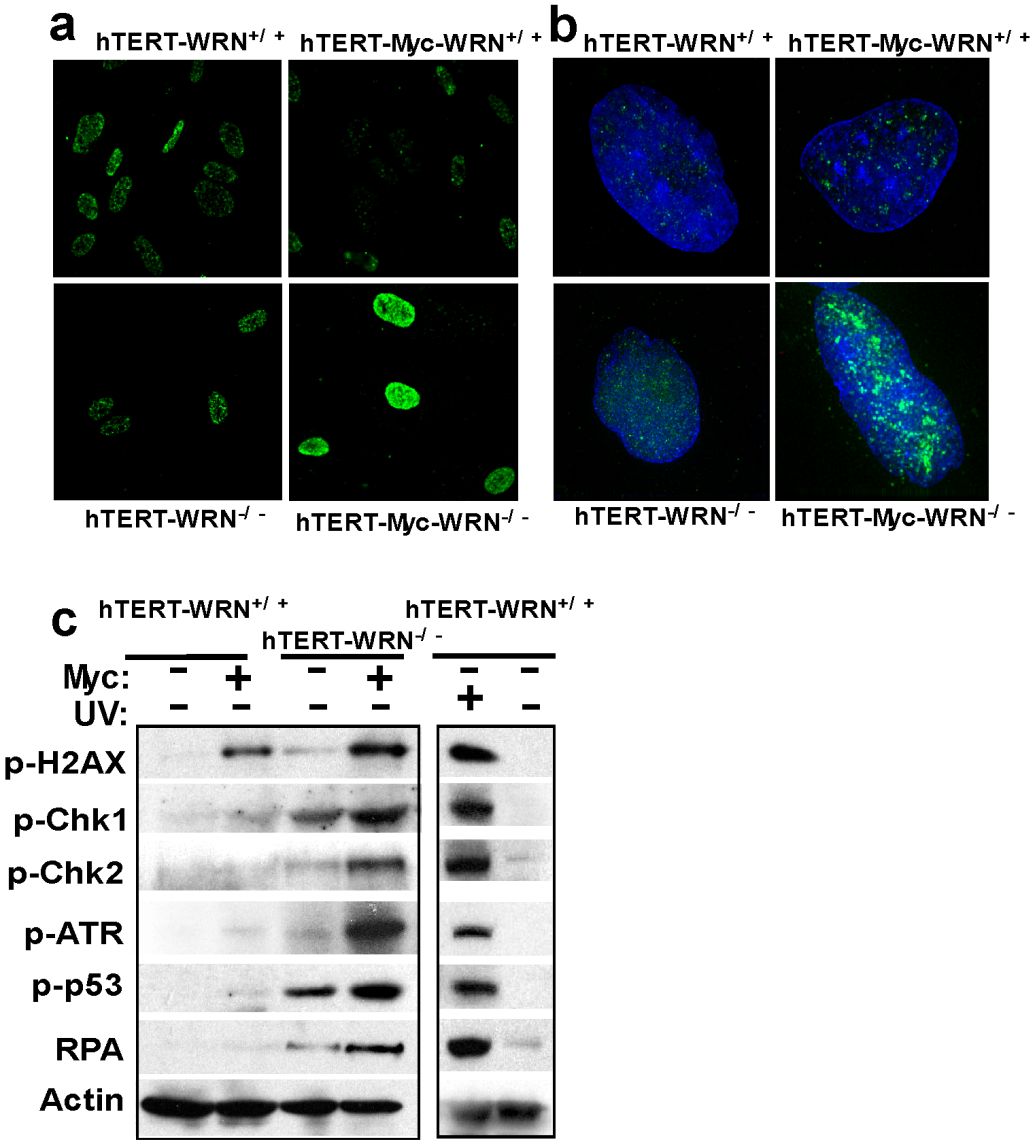
Replication stress induced in WRN deficient cells by c-Myc overexpression. a) *hTERT*-WRN^−/−^ cells or control *hTERT*-immortalized fibroblasts were transduced with LXSN-Myc and stained with anti-RPA (subunit 32) antibodies. Images were collected with a 40X oil–immersion objective. WRN^−/−^ cells expressing c-Myc showed prominent RPA nuclear staining. b) Cells as in (a) were subjected to a Triton extraction prior to fixation and stained for p-ATR (Ser428) to reveal the p-ATR associated with the chromatin resistant fraction. Images were collected with a 100X oil–immersion objective. Specific staining occurred in WRN^−/−^ cells upon c-Myc overexpression and was not detectable in the control *hTERT*–immortalized normal fibroblasts. c) Increased p-ATR (Ser428) and RPA (subunit 70) in WRN^−/−^ cells upon c-Myc overexpression was confirmed by Western analysis. In addition increased levels of p-CHK1, p-CHK2, and p-p53 by c-Myc were detected in WRN^−/−^ cells. HFFs exposed to UV are shown as positive control of DNA damage response.

The finding of DNA replication associated damage in c-Myc expressing WRN deficient cells suggested that intra-S checkpoints might be triggered. A first sensor of replication-associated damage is the single stranded DNA binding protein complex, RPA [27]. The RPA complex, which consists of three subunits, RPA70, 32 and 14, coats excess single strand DNA generated by aberrant or collapsed replication forks. Indeed, transduction of WRN^−/−^ cells with LXSN-Myc caused a striking increase in RPA nuclear speckles compared with c-Myc expressing WRN^+/+^ cells or relative to WRN^−/−^ cells transduced with an empty retroviral vector (Fig. 4a). RPA staining was increased in virtually every cell undergoing the first cellular division and thus likely during the first or second S-phase after c-Myc overexpression. However, RPA staining was not induced above background by LXSN-Myc transduction in fibroblasts expressing a functional WRN protein (Fig. 4a).

RPA is thought to signal activation of ATR and thereby trigger an intra-S checkpoint [27]. This leads to the tight association of ATR with chromatin surrounding the sites of damage [28]. Here we employed anti-phospho-ATR (Ser 428) specific antibody to stain the Triton-resistant nuclear fraction of cells, as an alternative to biochemical cell-fractionation techniques. Indeed this method has been employed to selectively detect relocalization of the Mre1 complex upon irradiation of cells [29]. WRN^−/−^ cells overexpressing c-Myc showed prominent phospho-ATR nuclear staining that was resistant to Triton extraction (Fig. 4b), confirming the activation of checkpoints by replication-associated damage. The increased level of p-ATR and RPA in c-Myc expressing WRN^−/−^ cells was confirmed by Western blot analysis (Fig. 4c).

DNA damage checkpoint signaling is mediated through the CHK1 and CHK2 kinases that, through phosphorylation of targets such as p53, CDC25A, and CDC25C, lead to cell cycle arrest [27]. Thus, we examined the levels of phospho- CHK1, CHK2, and p53 by Western blot analysis. The results shown in Fig. 4c indicate that indeed c-Myc overexpression led to increased phosphorylation of these downstream mediators selectively in WRN deficient cells, (Fig. 4c). These results, together with the increased accumulation of γ-H2AX foci at sites of newly replicated DNA, indicate the presence of replication stress. Thus, WRN is required to minimize replication-associated damage in response to c-Myc driven S-phase.

**Figure 5:**
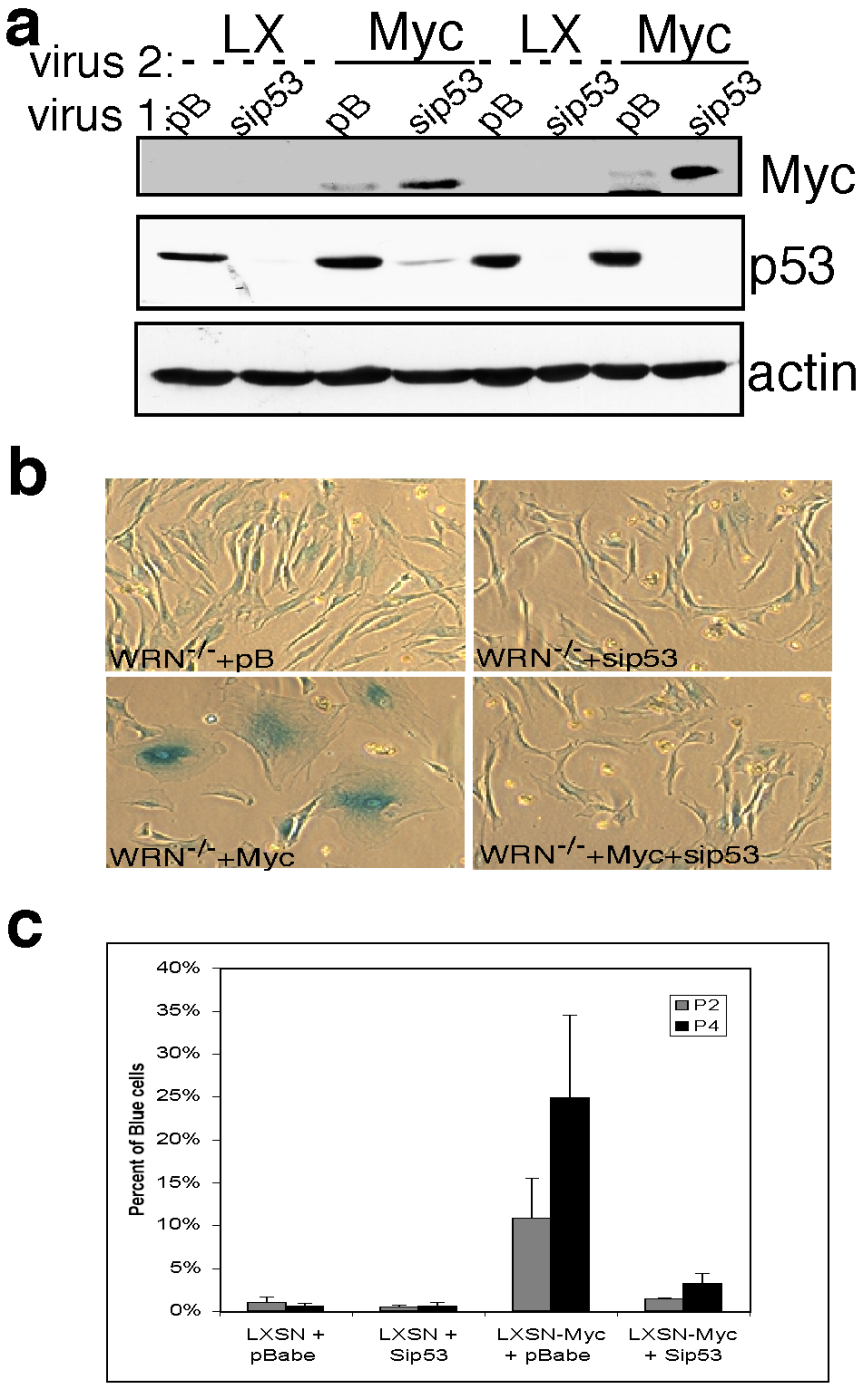
Knockdown of p53 rescues senescence in WRN^−/−^ cells in response to c-Myc overexpression. a) Western analysis of *hTERT*-WRN^−/−^ cells simultaneously transduced with c-Myc and sip53 retroviral vectors indicates p53 knockdown and c-Myc overexpression. b) A representative field of cells as in (a) stained for senescence associated β-Gal. Knockdown of p53 rescues senescence and decreases the percentage of senescent cells. c) Quantitation of cells as in (a) that stained positive for SA-β-Gal at passage 2 and 4 after viral transduction and selection.

p53 is required for the intra-S checkpoint activated by low doses of radiation [30]. This is consistent with our findings of increased phosphorylation of p53. To test if the activation of p53 consequent to replication-associated DNA damage in c-Myc expressing WRN^−/−^ cells was responsible for the senescence response seen in these cells [24], we utilized stable RNA interference to reduce p53 (sh-p53). Simultaneous transduction of WRN^−/−^ cells with c-Myc and sh-p53 expressing retroviruses led to their continuous proliferation and bypassing of senescence, as shown by the lack of β-galactosidase staining (SA β-Gal), when compared to WRN^−/−^ +c-Myc cells with intact p53 (Fig. 5b and 5c). Silencing of the major transcriptional target of p53, the Cdk inhibitor p21, also allowed proliferation and rescued senescence of the WRN^−/−^ cells expressing c-Myc (data not shown). These experiments indicate that DNA damage caused by c-Myc in WRN deficient cells ultimately leads to activation of p53, cell-cycle arrest and senescence. A role for p53 in mediating cell-cycle arrest in response to c-Myc activation has been shown previously, albeit independently of abrogation of DNA repair mechanisms [31]. This is contrast with our results, obtained in both primary HFFs and hTERT-immortalized fibroblasts, where cell-cycle arrest and senescence are only observed if WRN is depleted (this work and [24]). Possible explanations to this discrepancy are discussed below.

In summary, using primary human fibroblasts we have shown that c-Myc directly regulates S-phase duration. This was shown by both overexpression as well as loss of function experiments. The acceleration of S-phase caused by c-Myc leads to increased replication associated damage that is exacerbated by loss of WRN function, consistent with the role for WRN in recovery of damaged replication forks [12]. While the notion that c-Myc overexpression leads to accumulation of double strand breaks with consequent increased frequency of chromosomal rearrangements has been long known [32], [7], multiple underlying mechanisms have been proposed, one of which is increased generation of ROS [8], [9], [33] and [10]. However, it was recently shown that DNA damage in c-Myc overexpressing cells could accumulate independently of ROS [34]. Here, we propose that the dramatic acceleration of S-phase in c-Myc overexpressing cells leads to an accumulation of abnormal replication structures that, if left unrepaired because of WRN depletion, become sites of double stranded breaks. It is important to notice that in normal early passage HFFs, such as those used here, c-Myc constitutive overexpression leads to low level of DNA damage unless WRN is depleted. This is true both in early passage cells and upon c-Myc mediated immortalization, where no chromosomal translocations are generated (Benanti et al, 2007 and Fig. 4b). These observations differ from the rapid occurrence of chromatid breaks and chromosomal translocations detected by acute activation of Myc-ER [7]. We attribute this difference to both the cell type as well as to the constitutive versus conditional expression of c-Myc. Consistently, HFFs do not display cell-cycle arrest and senescence in response to either Ras or c-Myc expression [35], [21]. Finally, in these early passage HFFs, c-Myc induced DNA damage and senescence only occurs if there is interference with a DNA-repair pathway, indicating that it is likely that different tissue types and perhaps the age of the organism, may determine the outcome of oncogenic stimulation and to what extent senescence might be an effective barrier to cancer development *in vivo*.

Recent literature indicates that a hyper-replication phenotype leading to a DNA damage response and senescence is a common consequence to expression of activated *ras* and *mos* [36], [37]. However, the effect of these oncoproteins upon the rate of S-phase and the co-localization of damage with replication sites was not reported. Thus, we add a new facet to this work. c-Myc shortens the duration of S-phase, which is different from the previous notion of c-Myc causing accelerated entry into S, through simultaneous activation of G1 cyclins [38]. There are very few examples in the literature of genetic regulation of S-phase duration. Loss of function of Acf1, a chromatin assembly factor that influences nucleosome periodicity in Drosophila, leads to a fast S-phase transit [39]. Similarly, depletion of the histone linker H1 in slime mold also leads to a dramatic acceleration of S-phase, consistent with the observed inhibitory activity of chromatin packaging upon DNA replication [40]. These findings suggest that higher order chromatin structure influences replication dynamics and origin usage (reviewed by [41]). The possibility that c-Myc may exert its effect upon DNA replication though alteration of chromatin structure is intriguing in light of the role of c-Myc in recruiting histone acetylase complexes [42] and its direct binding to known replication origins [1]. For example, studies in Drosophila have shown that tethering histone acetylases to chromatin increases origin activity [43].

Finally, the finding that c-Myc sensitizes cells to replication-stress indicates that replication-coupled DNA repair might represent an Achilles' heel of c-Myc-driven tumors to which new therapeutic approaches might be developed.

## Methods

### Cell Culture and viral transductions

For cell cycle analysis shown in Fig. 1, 2 and 3, primary human foreskin fibroblasts (HFF) were generated from fresh neonatal human foreskins and infected with retroviruses within the first ten population doublings as described [21]. The TGR *c-myc* −/− rat fibroblast line and TGR parental cells, shown in Fig. 1b, were obtained from J. Sedivy's Laboratory [22]. The TGR *c-myc* −/− cells were reconstituted to express c-Myc by transduction with the retroviral vector pBabe-puro expressing human c-Myc. All cells were cultured in Dulbecco's Modified Eagles Medium (DMEM) supplemented with 10% FBS, penicillin and streptomycin. WRN deficient fibroblasts, shown in Fig. 4 and 5, immortalized with hTert as well as normal hTert-immortalized fibroblasts were previously described [24]. Overexpression of c-Myc in these cells was obtained using the retroviral vector pLXSN expressing human c-Myc. For UV irradiation, HFFs were exposed to 20 mJoules/cm^2^ for two minutes and then collected six hrs post treatment.

### Stable RNA interference and SA-β-galactosidase staining

Retroviral vectors pB-siWRN#1 and pB-sip53 were previously described [24]. pB-siWRN#2 contained a hairpin corresponding to the human WRN cDNA bps 2524–42 (Genbank #L76937): 5′-ggtccaacaatcatctact-3′. For the experiment shown in Fig. 5, cells were exposed to both LXSN-Myc and pB-si-p53 viruses and selected with G418 to allow selection of c-Myc overexpressing cells. At passage two and three after selection cells were stained for SA-β-galactosidase as described [44].

### FACS analysis and BrdU labeling

For the pulse chase experiments shown in Fig. 1 cells were synchronized by thymidine block (2.5 mM) for sixteen hours, released and labeled with 200 uM BrdU for 30 min (HFFs) or 50 uM for one hour (TGR cells), and either fixed immediately in 70% ethanol or chased in BrdU-free media and collected at the indicated times. Nuclei were isolated from fixed cells, stained with fluorescein isothiocyanate (FITC) conjugated anti-BrdU antibody (Becton Dickinson), and resuspended in 50 ug propidium iodide per ml. Nuclei were then analyzed on a FACS Scan instrument (Becton Dickinson), and cell cycle fractions were quantified with CellQuest software (Becton Dickinson).

### Western blotting

Whole-cell lysates for Western blotting were prepared by trypsinization of cells, followed by PBS wash, and then lysing cell pellets directly in SDS-sample loading buffer. Samples were normalized by using equivalent cell numbers. SDS-polyacrylamide gels were transferred to Immobilon-P membranes (Millipore). Blots were probed with antibodies to WRN (611169, BD PharMingen); p53 (Ab-6 OP43, Oncogene Research Products); c-Myc (Mab C-33, Santa Cruz); actin (I-19, Santa Cruz). All other antibodies were from Cell Signaling: anti-p-ATR (S-428 #2853S); anti-p-CHK1 (Ser 296, #2349); anti-p-CHK1 (Thr 68, #2661); anti-p-H2AX (Ser 139, #2577); anti-p-p53 (Ser 15, #9286); anti-RPA 70 (#2267).

### Immunofluorescence microscopy

Immunofluorescence was performed on cells grown on cover-slips (#1 1/2×18 mm). Cells were synchronized in S-phase by either growing to confluence and then fixing fourteen -sixteen hrs after re-plating (Fig. 3 and 4) or by Thymidine block (Fig. 2), release and labeling with BrdU (200 µM) for 30 min at the indicated times prior to fixation. Cells were fixed with 4% paraformaldehyde for fifteen minutes at room temperature, washed with PBS and permeabilized with ice cold MeOH/Acetone (1∶1) for 30 sec. After blocking with PBS containing 0.005% TWEEN-20 and 5% goat serum for 30 min at 37°C, cover-slips were incubated for one-two hrs at 37°C with the following antibodies: mouse anti-BrdU (SIGMA B-23531, in the presence of DNase I, 4 ul/ml, Sigma D-7291), rabbit anti-WRN rabbit (gift from Ashwini Kamath-Loeb), mouse anti-RPA-32 (#NA18, Calbiochem), rabbit anti-c-Myc (N-262, Santa Cruz), rabbit anti-p-ATR (S-428 #2853S, Cell Signaling), anti-γ-H2AX (#07164, Upstate). The anti-p-ATR was designed to recognize p-ser428, a phospho-site predicted by Scansite algorithm (Scansite@.mit.edu)

Verified as *in vivo* phosphor-site upon UV irradiation of cultured cells (Cell Signaling). Rhodamine or FITC-conjugated goat anti-rabbit IgG or goat anti-mouse IgG (Jackson Laboratories) were used as secondary antibodies at 1∶1000 in blocking buffer. For nuclear staining, cover-slips were incubated with Hoechst for five min, washed and mounted on glass slides with DAPCO (1,4-Diazobicyclo-(2,2,2) octane (Sigma), spectrophotometric grade glycerol (Kodak). 625 mg DAPCO, 22.5 ml spectroglycerol, 2.5 ml 1XPBS pH 8.6)). Staining of Triton resistant nuclear fractions with anti-p-ATR (Fig. 5) was accomplished by washing cells twice with ice-cold PBS, extraction in Cytoskeleton Buffer (10 mM PIPES pH 6.8, 100 mM NaCl, 300 mM sucrose, 3 mM MgCl2, 1 mM EGTA, 0.5% Triton X-100) for five minutes on ice, followed by two cold rinses in PBS prior to fixing with 4% paraformaldehyde fifteen minutes. Staining was then carried on as above. Confocal images (stacks) were acquired at 0.2 micron spacing with an Olympus 100× or a 40× Oil immersion objective as specified in the figure legends with an Applied Precision DeltaVision RT microscope system (Applied Precision, Issaquah, WA). The exposure times were kept constant for each fluorescence channel within each experiment and antibody used. Stacks were deconvolved using a constrained iterative algorithm with DeltaVision SoftWorx software.

## Supporting Information

Movie S1 Co-localization of WRN and BrdU in HFF-Myc. HFF-Myc synchronized by double thymidine block were released in S phase and labeled after 1 hr with BrdU for 30′prior to fixation. WRN and BrdU were detected with specific antibodies followed by a FITC or Rhodamine conjugate secondary, respectively. Yellow spots present throughout the nucleus indicate overlap of WRN protein with newly replicated DNA. Images were collected using an Olympus 100x immersion objective with an Applied Precision DeltaVision RT microscope system (Applied Precision, Issaquah, WA). 3D reconstruction was obtained using Imaris software (Bitplane).
(12.35 MB MOV)

Movie S2 Predominant nucleolar localization of WRN in HFF-pB. HFF-pB were synchronized and processed as for Movie 1. In these cells WRN staining (green) is shown mostly in nucleoli and only a few extra-nucleolar sites coincide with BrdU.
(10.18 MB MOV)

Figure S1 Cell-cycle profile of HFF-pB and HFF-Myc after Thymidine block. Hffs, synchronized by double thymidine block, were stained with Propidium iodide and analyzed by FACS. Both cell populations HFF-pB and HFF-Myc show an arrest at the G1/S boundary. HFF's exponentially growing cell cycle profiles are shown as reference.
(4.80 MB TIF)
