# Supplementary figures and images for: c-Myc Accelerates S-Phase and Requires WRN to Avoid Replication Stress
Source: PLoS One. 2009 Jun 18;4(6):e5951. doi: 10.1371/journal.pone.0005951 (PMC2694031; doi:10.1371/journal.pone.0005951)

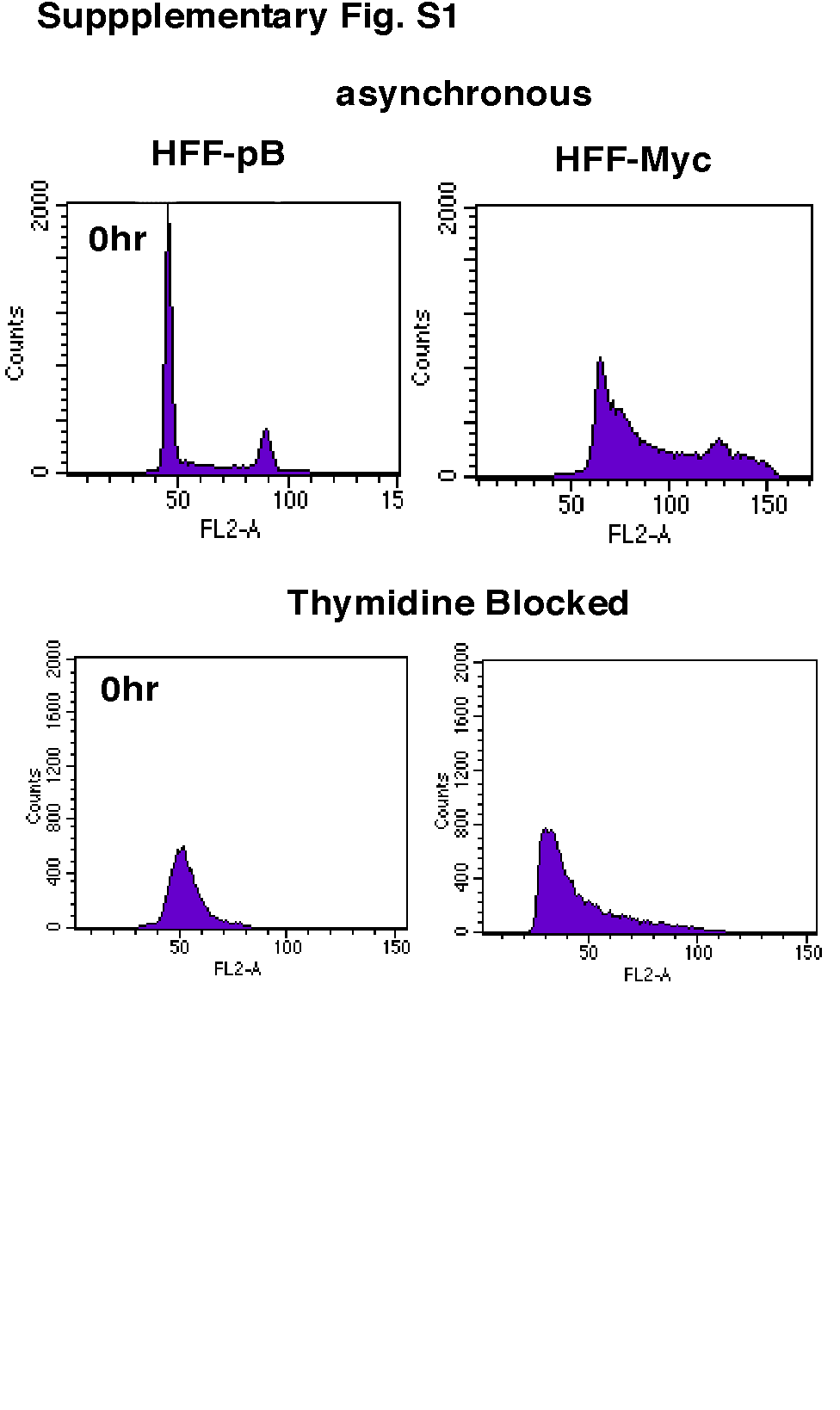

Supplement: Figure S1 — Cell-cycle profile of HFF-pB and HFF-Myc after Thymidine block. Hffs, synchronized by double thymidine block, were stained with Propidium iodide and analyzed by FACS. Both cell populations HFF-pB and HFF-Myc show an arrest at the G1/S boundary. HFF's exponentially growing cell cycle profiles are shown as reference. (4.80 MB TIF) [file pone.0005951.s003.tif]
